# Branchial Cleft Cyst Carcinoma Remains Grossly Over Diagnosed: A Large Database Analysis

**DOI:** 10.1002/lary.70060

**Published:** 2025-08-22

**Authors:** Andrew Meci, Neerav Goyal, David Goldenberg

**Affiliations:** ^1^ The Pennsylvania State University College of Medicine Hershey Pennsylvania USA; ^2^ Department of Otolaryngology Penn State Milton S. Hershey Medical Center Hershey Pennsylvania USA

**Keywords:** branchial cleft cyst, branchial cleft cyst carcinoma, cohort study, database, malignant branchial cleft cyst

## Abstract

**Introduction:**

Branchial cleft cyst carcinomas (BCCC) are rare lateral neck malignancies thought to originate from branchial cleft remnants. Diagnosing primary BCCC should be approached with skepticism; cystic nodal metastasis must be excluded. Due to its rarity, we performed a comprehensive database study to better characterize the ongoing diagnosis of BCCC.

**Methods:**

Single‐arm retrospective cohort study using the TriNetX Research database. Patients ≥ 18 years of age with a diagnosis of BCCC identified by ICD‐10 C10.4 between 2008 and 2018 were included, allowing for up to 5 years of follow‐up. Demographics, past medical history, oncologic history, diagnostic rate, treatment pathways, and 5‐year outcomes including diagnosis with primary HNC and Kaplan–Meier survival curves were analyzed within TriNetX.

**Results:**

In 10 years, 1070 patients diagnosed with BCCC were included. The mean age was 59.6 ± 9.7; the majority were male (*n* = 810, 75.6%) and white (*n* = 765, 71.4%). Incidence of BCCC increased from 2008 to 2015 and subsequently dropped precipitously. Almost all patients, 94.4% (*n* = 1011), had prior or subsequent diagnosis of another HNC within 5 years. Five‐year survival probability for BCCC was 56.0%.

**Conclusions:**

We describe the largest cohort of BCCC patients to date. Most patients were diagnosed with another HNC within 5 years, suggesting a likely misdiagnosis of BCCC. A poor 5‐year survival rate may be secondary to a delay in appropriate treatment following an erroneous diagnosis, underscoring the need to approach a diagnosis of BCCC with caution. We consider the use of the BCCC code to be detrimental and recommend its discontinuation.

**Level of Evidence:**

4.

## Graphical abstract

Branchial cleft cyst carcinomas (BCCC) are rare lateral neck malignancies thought to originate from branchial cleft remnants. This study describes the largest cohort of patients with BCCC to date. Most patients were diagnosed with another HNC within 5 years, suggesting a likely misdiagnosis of BCCC.

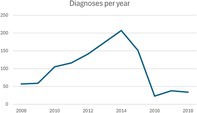

## 1 Introduction

Branchial cleft cyst carcinomas (BCCC) are extremely rare lateral cervical cystic malignancies theorized to arise from malignant transformation of branchial cleft remnants [1]. However, since the branchial theory of lateral cervical cyst development by Ascherson in 1832, the diagnosis of branchial cleft cyst has remained contested [2] and, by extension, the diagnosis of branchial cleft cyst carcinoma theoretical [3, 4]. Instead, it has been proposed that lateral cervical cysts arise from cystic degeneration of lymph nodes and in fact represent malignant adenopathy [2, 4].

Case reports of primary branchial cleft cyst carcinoma continue to be published in the literature [5, 6] according to stringent diagnosis initially established by Martin et al. and later expanded upon by Khafif et al. [3, 7] These authors argue that primary branchial cleft cyst carcinomas are poorly understood entities due to their rarity [8]. Fewer than 40 reports of BCCC diagnosis exist in the literature [1].

Given the persistence of controversy surrounding BCCC in the literature and its rarity, a large claims database could be of use in characterizing branchial cleft cyst carcinomas and in distinguishing this condition from other head and neck malignancies. We present the largest study to date investigating the diagnosis of branchial cleft cyst carcinoma.

## 2 Materials and Methods

### 2.1 Data Source and Study Design

The study data was obtained from the TriNetX Research Network (Cambridge, MA) on August 1, 2024. TriNetX is a global federated health research network providing access to electronic medical records (diagnoses, procedures, medications, laboratory values) from large healthcare organizations (HCOs) [9]. Mortality data is sourced from HCO electronic health records in addition to Social Security Administration and federal death registry data. The TriNetX platform only uses aggregated counts and statistical summaries of de‐identified information. No protected health information or personal data is available to the platform users. Therefore, this study was deemed exempt by the Penn State University Institutional Review Board (IRB), and the need for informed consent was waived (STUDY18629). This study follows the Strengthening the Reporting of Observational Studies in Epidemiology (STROBE) reporting guidelines for cohort studies.

### 2.2 Participants

The database was queried on August 1, 2024, to identify patients ≥ 18 years of age who were diagnosed with BCCC between January 1, 2008, and December 31, 2018, allowing for up to 5 years of follow‐up. BCCC was defined as either ICD‐O C10.4 or IDC‐10‐CM C10.4: malignant neoplasm of the branchial cleft.

### 2.3 Outcome Measures

Cohort demographics, past medical history, and oncologic information were obtained, as well as analysis of annual incidence from 2008 to 2018. Demographic measures included age, sex, race, and ethnicity. The annual incidence of BCCC diagnosis and concurrent diagnosis of head and neck cancer malignancies and symptomologies was recorded. Concurrent diagnoses were not described exclusively, and overlap of diagnoses was allowed (Table S1). Tumor‐node‐metastasis staging data was also collected. Diagnosis and treatment patterns were recorded for 1 year prior to diagnosis and 5 years after diagnosis. These procedures included imaging, endoscopic studies, fine needle aspiration, excisional biopsy, chemotherapy, radiation, and excisional surgery (Table S2).

All analyses were conducted on the TriNetX platform [10]. Numerical variables were summarized as means and standard deviations, while categorical variables were expressed as n and proportion of the total. TriNetX offers tools to determine lines and timing of treatment as well as arrival rates and incidences of diagnoses. Kaplan–Meier curves were used to determine 5‐year survival. One‐armed risk analyses of later diagnosis with a different head and neck cancer were reported as a risk percentage.

## 3 Results

**FIGURE 1:**
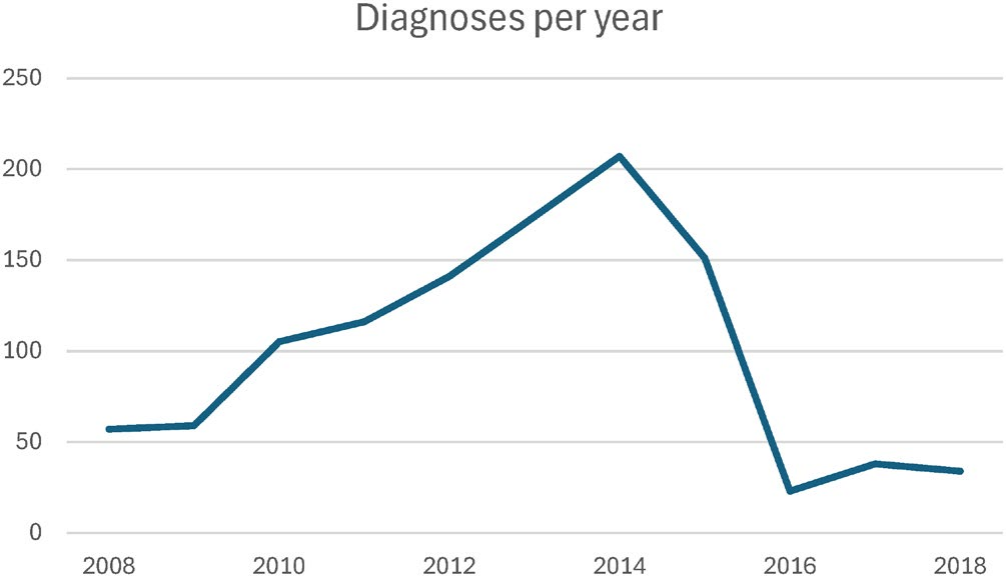
Branchial cleft cyst carcinomas (BCCC) are rare lateral neck malignancies thought to originate from branchial cleft remnants. This study describes the largest cohort of patients with BCCC to date. Most patients were diagnosed with another HNC within five years, suggesting a likely misdiagnosis of BCCC. [Color figure can be viewed in the online issue, which is available at www.laryngoscope.com]

Among 1071 patients diagnosed with BCCC between January 1, 2008, and December 31, 2018, the mean age was 59.6 ± 9.7; patients were 75.6% male (*n* = 810), 71.4% white (*n* = 765), and just 4.0% were Hispanic or Latino (*n* = 43) (Table 1). The overall mean annual incidence rate of BCCC was 0.072 per 100,000 person‐years. The rate of diagnosis was variable during the study period, with an increase of diagnoses/year from 2008 to 2015, with a precipitous drop between 2015 and 2016 and then a slight increase of diagnosis/year between 2016 and 2018 (Figure 1).

**TABLE 1:** Population demographics of patients diagnosed with BCCC between 2008 and 2018.

Age	59.6 ± 9.7
Sex, *n* (%)	
Male	810 (75.6%)
Female	261 (24.4%)
Race, *n* (%)	
White	765 (71.4%)
Black or African American	121 (11.3%)
Asian	51 (4.8%)
Native Hawaiian or Other Pacific Islander	< 10 (< 1.0%)
Native American or Alaskan	< 10 (< 1.0%)
Other	17 (1.6%)
Unknown	112 (10.5%)
Ethnicity, *n* (%)	
Hispanic or Latino	43 (4.0%)
Not Hispanic or Latino	664 (62.1%)
Unknown	364 (34.0%)

Among included patients, 24.5% (*n* = 263) had lymphadenopathy, 12.6% (*n* = 135) had malaise or fatigue, 11.0% (*n* = 118) had abnormal weight loss, 41.7% (*n* = 447) had aphagia or dysphagia, and 40.8% (*n* = 437) had a localized subcutaneous mass prior to diagnosis with BCCC (Table 2). Diagnostic testing prior to diagnosis included imaging studies (46.2%, *n* = 495), laryngeal endoscopy (45.7%, *n* = 45.7), fine needle aspiration (11.8%, *n* = 126), and excisional biopsy or surgery (59.7, *n* = 639) (Table 3).

**TABLE 2:** Oncologic features and treatment of patients diagnosed with branchial cleft cyst carcinoma between 2008 and 2018.

HNC symptoms, *n* (%)	
Lymphadenopathy	263 (24.6%)
Malaise or fatigue	135 (12.6%)
Abnormal weight loss	118 (11.0%)
Aphagia or dysphagia	447 (41.7%)
Fever of unknown origin	95 (8.9%)
Localized mass	437 (40.8%)
Any HNC diagnosis prior to BCCC	815 (76.1%)
Malignant head and neck neoplasm of unknown primary at time of BCCC diagnosis	67 (6.3%)
HNC Diagnoses (prior to or within 5 years after BCCC diagnosis), *n* (%)	1011 (94.4%)
Oral cavity	596 (55.6%)
Unspecified parts of tongue	371 (34.6%)
Oropharynx	956 (89.3%)
Tonsil	439 (41.0%)
Base of tongue	373 (34.8%)
Other sites	953 (90.0%)
Hypopharynx	115 (10.7%)
Larynx	205 (19.1%)
Secondary neoplasm of head/neck lymph nodes	365 (34.0%)
Secondary neoplasm of unspecified site	273 (25.5%)
HPV+, *n* (%)	41 (3.8%)
T staging, *n* (%)	313 (29.2%)
TX	104 (9.7%)
T1	72 (6.7%)
T2	85 (7.9%)
T3	61 (5.7%)
T4	101 (9.4%)
N staging, *n* (%)	308 (28.8%)
NX	90 (8.4%)
N0	116 (10.8%)
N1	61 (5.7%)
N2	150 (14.0%)
N3	15 (1.4%)
M staging, *n* (%)	270 (25.2%)
M0	252 (23.6%)
M1	21 (2.0%)

Abbreviations: BCCC, branchial cleft cyst carcinoma; HNC, head and neck cancer.

**TABLE 3:** Diagnostic and treatment patterns of patients diagnosed with branchial cleft cyst carcinoma (BCCC) between 2008 and 2018. Time periods were 1 year before BCCC diagnosis and 5 years after BCCC diagnosis.

Diagnostic testing/treatment before BCCC diagnosis	
Imaging studies	543 (50.7%)
Laryngeal endoscopy	489 (45.7%)
With biopsy	169 (15.8%)
Esophageal endoscopy	208 (19.4%)
Fine needle aspiration	126 (11.8%)
Excisional biopsy	16 (1.5%)
Excisional surgery	219 (20.4%)
Neck dissection	20 (1.9%)
Tonsillectomy	48 (4.5%)
Glossectomy	58 (5.4%)
Chemo/radiation therapy	555 (51.8%)

Diagnostic testing/treatment after BCCC diagnosis	
Imaging studies	595 (55.6%)
Laryngeal endoscopy	444 (41.5%)
With biopsy	116 (10.8%)
Esophageal endoscopy	206 (19.2%)
Fine needle aspiration	103 (9.6%)
Excisional biopsy	< 10 (1.0%)
Excisional surgery	165 (15.4%)
Neck dissection	61 (5.7%)
Tonsillectomy	16 (1.5%)
Glossectomy	37 (3.5%)
Radiation therapy (alone)	129 (12.0%)
Chemo/radiation therapy	578 (54.0%)

The majority of patients, 76.1% (*n* = 815), had diagnoses of head and neck cancer (HNC) prior to BCCC diagnosis. Of the 256 patients whose initial diagnosis of HNC was BCCC, 196 were diagnosed with another form of HNC within the 5 years. This amounts to 94.4% of patients having a prior or subsequent HNC diagnosis. Among BCCC patients, co‐diagnoses of head and neck cancer were primarily located in the oropharynx (*n* = 956, 89.3%), with 41.8% (*n* = 439) in the tonsils and 34.8% (*n* = 373) in the base of tongue. Other prominent sites of malignancy included the oral cavity (*n* = 596, 55.6%), hypopharynx (*n* = 115, 10.7%), and larynx (*n* = 205, 19.1%). There were 67 (6.3%) patients who held a diagnosis of malignant neoplasm of unknown primary of the head and neck at the time they were diagnosed with BCCC. There were 365 (34.0%) patients who were diagnosed with local or distant lymphatic metastasis of the head and neck region (Table 2). Available TNM staging data were limited to under 30% of the total cohort, with the most common overall stage being Stage 4 (17.9%, *n* = 192) (Table 2).

Diagnostic procedures prior to the diagnosis of BCCC included imaging of the head and neck (*n* = 543, 50.7%), laryngoscopy (*n* = 489, 45.7%), and fine needle aspiration (*n* = 126, 11.8%). Of patients diagnosed with BCCC, 15.4% (*n* = 165) underwent excisional surgery, 54.0% (*n* = 598) underwent chemo‐radiation therapy, and 12.0% (*n* = 129) underwent radiotherapy alone (Table 3).

**FIGURE 2:**
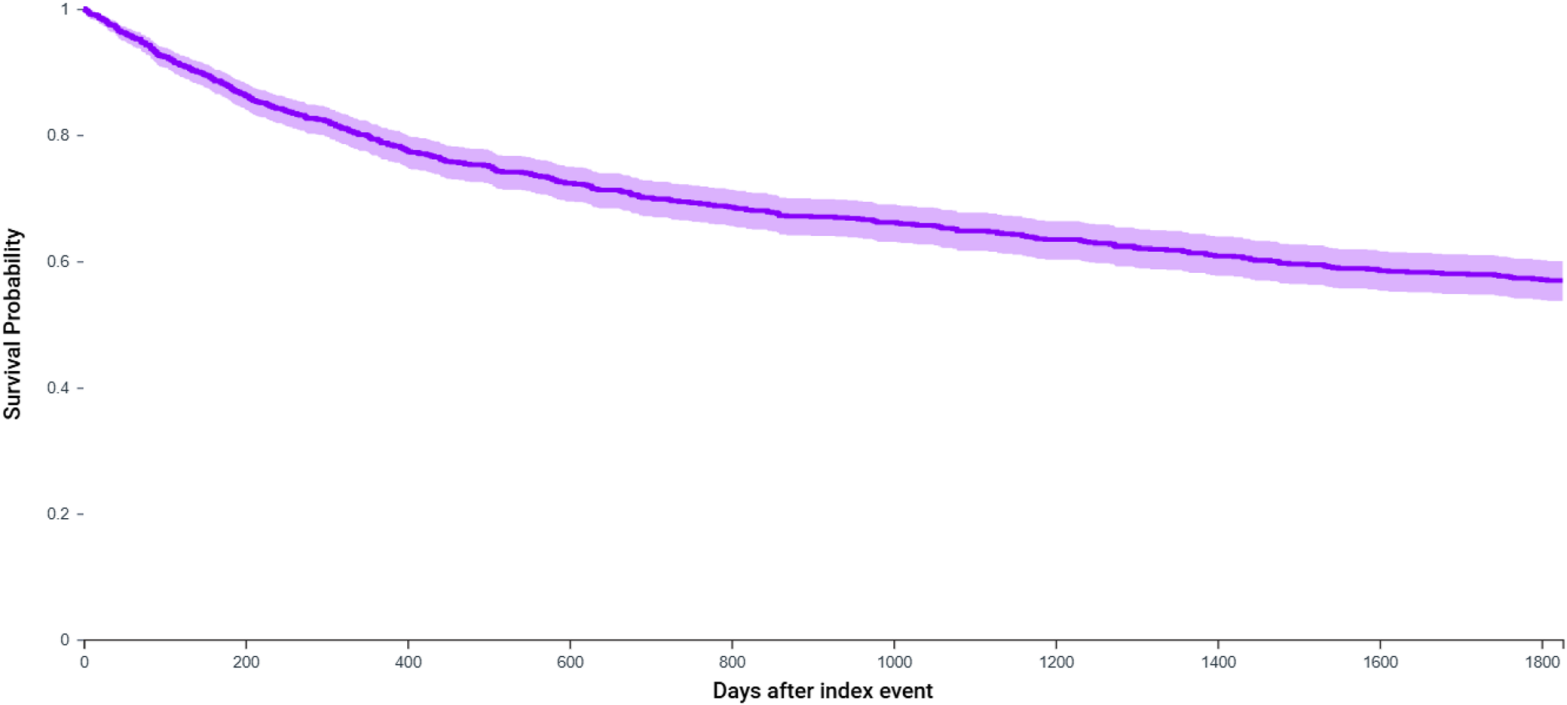
Five‐year survival curve for patients diagnosed with branchial cleft cyst carcinoma. Survival probability is 56.9% at 5 years. [Color figure can be viewed in the online issue, which is available at www.laryngoscope.com]

Of patients who received treatment, surgery was typically first‐line (46.8%; *n* = 501) and within 74 days of diagnosis (±215 days). BCCC patients had a five‐year survival probability of 56.0% (*n* = 401; 37.5% deceased) (Figure 2).

## 4 Discussion

Our study aimed to characterize BCCC diagnoses over 10 years and with at least 5 years of follow‐up. This investigation analyzed the largest cohort of patients with a diagnosis of branchial cleft cyst carcinoma using a large database analysis. We find that despite controversy regarding the existence of this disease process, patients continue to receive this diagnosis. The overwhelming majority of patients in our study had a prior diagnosis of head and neck cancer (76.2%) and, if not, had a high risk of later diagnosis with HNC (76.8%), resulting in 94.5% having a concurrent diagnosis of HNC within 5 years of BCCC diagnosis. This casts doubt that BCCC is indeed a primary tumor.

**FIGURE 3:**
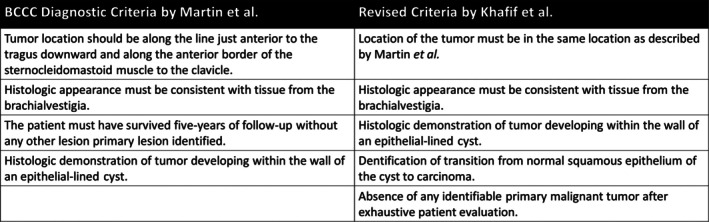
Branchial cleft cyst carcinoma diagnostic criteria.

Von Volkmann in 1882 was the first to posit that some malignant cervical tumors may arise from branchial cleft remnants [11]. However, this proposition was only based on the fact that he could not find primary lesions in the oral cavity or pharynx. Even during that period, this origin was disputed by authors like Sutton, who in 1893 contended that these masses were secondary to epithelioma originating in the oropharynx or nasopharynx [4]. Despite this dissent and a lack of histologic evidence to support the diagnosis, BCCC remained a utilized and treated diagnosis. BCCC was most concretely validated with a landmark paper by Hayes Martin et al., in which the authors established four stringent criteria for diagnosis (Figure 3) [3]. It is important to note that by using these criteria to review new and previously described cases of BCCC, the authors were unable to confirm even one instance that satisfied all four criteria, leading to the conclusion that branchiogenic cancer is an entirely hypothetical diagnosis [3, 4]. Khafif et al. in 1989, finding few patients that filled these criteria, especially with respect to the third criterion of Martin et al., suggested removing the third criterion and adding two additional criteria: (1) identification of the transition of normal squamous epithelium of the cyst to carcinoma and (2) absence of any identifiable primary malignant tumor after exhaustive evaluation of the patient (Figure 3) [4, 5, 7]. Since the publication of these two landmark papers, less than 40 reported cases have been published claiming to fit the criteria [1], and other methodologies have been proposed to identify BCCC [12]. Despite the small number of reported cases of BCCC, our database analysis found over 1000 diagnoses of BCCC within 10 years, seemingly disproportionate to the exceeding rarity of the condition and the stringency of established criteria.

Ascherson (1832), in his “branchial theory,” suggested that incomplete obliteration of branchial cleft mucosa remains dormant until stimulated to grow later in life, resulting in cyst formation [13]. A recent review has found no evidence to support Ascherson's branchial cleft cyst theory of lateral cervical cyst development, which outright refutes the existence of branchial cleft cyst carcinoma [4]. Instead, there is substantial evidence to support the theory that lateral cervical cysts form due to lymph node cystic transformation stimulated by the inclusion of tonsillar crypt epithelium during infant or adult life [2, 14, 15]. Further study has supported a theory of cystic metastasis from primary SCC in Waldeyer's ring, which has abundant vascular and lymphatic supply and may lead to early nodal metastasis with even small indolent oropharyngeal primary malignancy [2, 16, 17, 18]. Human papillomavirus (HPV) preferentially targets reticulated tonsillar crypt epithelium, which suggests HPV infection could be related to cystic node metastasis [19, 20, 21]. Our study findings support these theories, with evidence that co‐diagnoses of head and neck cancer are primarily located in the oropharynx (89.3%), with 41.8% and 34.8% of malignancies in the tonsils and base of tongue. Further, 44.0% were found to have a secondary neoplasm of the lymph nodes. TriNetX database does not accurately record HPV data, and therefore we were unable to assess the prevalence of HPV infection in our cohort.

Given the rarity of BCCC and the cystic presentation of HPV‐induced cystic lymph node metastasis, it can be supposed that many in our cohort were erroneously diagnosed with BCCC. Insufficient or inappropriate patient care is the primary risk of carrying a misdiagnosis of branchial cleft cyst carcinoma. This is highlighted by low proportions undergoing imaging studies (50.7%), laryngoscopy (45.7%), and fine needle aspiration (11.8%) prior to BCCC diagnosis. We found that 20.4% of patients had an excisional surgery in the year prior to diagnosis with BCCC, and that 15.4% were treated with excisional surgery of the head and neck in the 5 years following diagnosis. Further, we found that only 1.9% and 5.7% of patients underwent a neck dissection before and after BCCC diagnosis. Violation of neck tissues from an excisional biopsy when a neck dissection would be the proper treatment may subsequently require more extensive surgery and increase patient morbidity and mortality [4, 22, 23, 24, 25]. These findings may explain the relatively poor five‐year overall survival of 56.0% for BCCC in our study.

One potential theory for undertreatment or insufficient investigation may be the existence of the BCCC code itself. BCCC is extremely rare, and treatment of the condition as a primary tumor when there is likely a primary cancer located elsewhere in Waldeyer's ring could have devastating impacts on patient outcomes. It is plausible that this code is being used as a placeholder while the investigation for a primary tumor is conducted. However, it is also possible that clinicians are overusing the condition due to a lack of knowledge of its strict diagnostic criteria. While there has been discussion of improper psychiatric diagnoses impacting the care of racial and ethnic groups, there have been no studies to date positing this phenomenon as an influence on oncological care [26]. Of note, we did find a sharp drop in BCCC diagnoses after 2014; though analysis of more recent data has revealed that diagnosis rates have again begun to grow. We do not fully understand why diagnoses fell after 2014, as diagnostic criteria and coding structures for BCCC did not change at that time. Regardless, given the impact of potential improper oncologic care, our study supports Martin's Rule that an adult patient with a solitary lateral cystic neck mass should be presumed to have a cystic lymph node metastasis until proven otherwise, and that there must be prompt investigation for the primary cancer [3].

Despite the strengths and findings of our investigation, important limitations exist within the context of our study question and design. While the TriNetX network is expansive, there is limited oncologic data related to staging, especially HPV status. We are also limited in our ability to examine patient diagnostic workup and treatment, as 4.0% of patients were not included in the TriNetX dataset prior to their BCCC diagnosis and 1.5% did not have follow‐up visits or treatment documented. Additionally, these limitations may have led to undercounts of prior HNC diagnoses in this cohort of patients. As with all large electronic medical record‐sourced database studies, our analysis is also limited by the accuracy of data entry.

## 5 Conclusions

We describe the largest cohort of BCCC patients to date. Although there is controversy surrounding the existence of BCCC, our analysis indicates that the ICD‐10 code continues to be improperly utilized, as almost all patients are being diagnosed with another HNC within 5 years. A poor five‐year survival rate may be secondary to a delay in appropriate treatment following an erroneous diagnosis, underscoring the need to be skeptical of a BCCC diagnosis. A solitary lateral cystic neck mass should be considered a cystic lymph node metastasis until proven otherwise, and a search for the primary cancer is essential.

## Conflicts of Interest

The authors declare no conflicts of interest.

## Supporting information


**Table S1:** Coding for head and neck malignancy locations.
**Table S2:** Coding for diagnostic and treatment patterns.

## Data Availability

The data that support the findings of this study are available from the corresponding author upon reasonable request.
