# Supplementary material for: Branchial Cleft Cyst Carcinoma Remains Grossly Over Diagnosed: A Large Database Analysis
Source: Laryngoscope. 2025 Aug 22;136(2):766–72. doi: 10.1002/lary.70060 (PMC12793964; doi:10.1002/lary.70060)
Supplement: Supplementary file 1 — Table S1: Coding for head and neck malignancy locations. Table S2: Coding for diagnostic and treatment patterns. [file LARY-136-766-s001.docx]

**Supplemental Table 1:** Coding for head and neck malignancy locations.

| **HNC location** | **Code system** | **Included codes** | **Included code description** | **Excluded codes** | **Excluded code description** |
| --- | --- | --- | --- | --- | --- |
| Oral Cavity | ICD-10-CM | C00 | Malignant neoplasm of lip | C00.0 | Malignant neoplasm of external upper lip |
|  | ICD-10-CM | C02 | Malignant neoplasm of other and unspedified parts of tongue | C00.1 | Malignant neoplasm of external lower lip |
|  | ICD-10-CM | C03 | Malignant neoplasm of gum | C00.2 | Malignant neoplasm of external lip, unspecified |
|  | ICD-10-CM | C04 | Malignant neoplasm of floor of mouth |  |  |
|  | ICD-10-CM | C05 | Malignant neoplasm of palate |  |  |
|  | ICD-10-CM | C06 | Malignant neoplasm of other and unspecified parts of mouth |  |  |
| Oropharynx | ICD-10-CM | C01 | Malignant neoplasm of base of tongue | C10.4 | Malignant neoplasm of branchial cleft |
|  | ICD-10-CM | C09 | Malignant neoplasm of tonsil |  |  |
|  | ICD-10-CM | C10 | Malignant neoplasm of oropharynx |  |  |
| Hypopharynx | ICD-10-CM | C13 | Malignant neoplasm of hypopharynx |  |  |
| Larynx | ICD-10-CM | C32 | Malignant neoplasm of larynx |  |  |
| Secondary Site | ICD-10-CM | C79.9 | Secondary malignant neoplasm of unspecified site |  |  |
| Secondary Lymph Nodes | ICD-10-CM | C77.0 | Secondary and unspecified malignant neoplasm of lymph nodes of head, face and neck |  |  |

**Supplemental Table 2:** Coding for diagnostic and treatment patterns.

| **Diagnostic Test or Treatment** | **Code system** | **Code** | **Description** |
| --- | --- | --- | --- |
| Imaging Studies | CPT | 1010253 | Diagnostic radiology procedures of the head and neck |
|  | CPT | 1010760 | Diagnostic ultrasound procedures of the head and neck |
| Laryngeal endoscopy | CPT | 1005837 | Endoscopy procedures on the larynx |
| Laryngeal endoscopy with biopsy | CPT | 1014609 | Laryngoscopy, direct, operative, with biopsy |
| Esophageal endoscopy | CPT | 1007241 | Endoscopy procedures on the esophagus |
| Fine needle aspiration | CPT | 38505 | Biopsy or excision of lymph nodes; by needle, superficial |
|  | CPT | 1003145 | Fine needle aspiration (depreciated 2020) |
|  | CPT | 1035129 | Fine needle aspiration biopsy procedures |
| Excisional biopsy | CPT | 1006903 | Limited lymphadenectomy for staging procedures |
|  | CPT | 38500 | Biopsy or excision of lymph nodes; open, superficial |
|  | CPT | 38505 | Biopsy or excision of lymph nodes; open, deep cervical nodes |
|  | CPT | 38520 | Biopsy or excision of lymph nodes; open, deep cervical nodes with excision of scalene fat pad |
|  | CPT | 21550 | Biopsy soft tissue of neck or thorax |
| Excisional surgery | CPT | 1005695 | Excision procedures on the nose |
|  | CPT | 1005743 | Destruction procedures on the nose |
|  | CPT | 1005780 | Excision procedures on the accessory sinuses |
|  | CPT | 1005815 | Excision procedures on the larynx |
|  | CPT | 1005879 | Destruction procedures on the larynx (deprecated 2022) |
|  | CPT | 1006891 | Excision procedures on the lymph nodes and lymphatic channels |
|  | CPT | 1006903 | Limited lymphadenectomy for staging procedures |
|  | CPT | 1006913 | Radical lymphadenectomy (radical resection of lymph nodes) |
|  | CPT | 1006966 | Excision procedures on the lips |
|  | CPT | 1006997 | Excision and destruction procedures on the vesituble of the mouth |
|  | CPT | 1007034 | Excision procedures on the tongue and floor of mouth |
|  | CPT | 1007071 | Excision and destruction procedures on the dentoalveolar structures |
|  | CPT | 1007091 | Excision and destruction procedures on the palate and uvula |
|  | CPT | 1007133 | Excision procedures on the tongue and floor of mouth |
|  | CPT | 1007168 | Excision and destruction procedures on the pharynx, adenoids, and tonsils |
|  | CPT | 1007219 | Excision procedures on the esophagus |
|  | CPT | 1009028 | Excision procedures on the thyroid gland |
|  | CPT | 1003998 | Excision, tumor, soft tissue of neck or anterior thorax, subcutaneous |
|  | CPT | 1020176 | Excision, tumor, soft tissue of neck or anterior thorax, subfascial |
| Neck dissection | CPT | 38700 | Suprahyoid lymphadenectomy |
|  | CPT | 38720 | Cervical lymphadenectomy (complete) |
|  | CPT | 38724 | Cervical lymphadenectomy (modified radical neck dissection) |
| Tonsillectomy | CPT | 1007178 | Tonsillectomy and adenoidectomy |
|  | CPT | 1007190 | Radical resection of tonsil, tonsillar pillars, and/or retromolar trigone |
|  | CPT | 1007181 | Tonsillectomy, primary or secondary |
| Glossectomy | CPT | 1007040 | Excision of tongue with closure |
|  | CPT | 1007046 | Glossectomy |
| Chemotherapy | TNX Curated | 1002 | Chemotherapy |
| Radiotherapy | TNX Curated | 1001 | Radiation |
